# HLA-F Allele-Specific Peptide Restriction Represents an Exceptional Proteomic Footprint

**DOI:** 10.3390/ijms20225572

**Published:** 2019-11-08

**Authors:** Gia-Gia T. Hò, Funmilola J. Heinen, Rainer Blasczyk, Andreas Pich, Christina Bade-Doeding

**Affiliations:** 1 Institute for Transfusion Medicine, Hannover Medical School, Carl-Neuberg-Str. 1, 30625 Hannover, Germany; Ho.Gia-Gia@mh-hannover.de (G.-G.T.H.); funmilola.heinen@mpibpc.mpg.de (F.J.H.); Blasczyk.Rainer@mh-hannover.de (R.B.); 2 Institute of Toxicology, Hannover Medical School, Carl-Neuberg-Str. 1, 30625 Hannover, Germany; Pich.Andreas@mh-hannover.de

**Keywords:** HLA-F, peptides, peptide selection, proteome

## Abstract

Peptide-dependent engagement between human leucocyte antigens class I (HLA-I) molecules and their cognate receptors has been extensively analyzed. HLA-F belongs to the non-classical HLA-Ib molecules with marginal polymorphic nature and tissue restricted distribution. The three common allelic variants HLA-F*01:01/01:03/01:04 are distinguished by polymorphism outside the peptide binding pockets (residue 50, α1 or residue 251, α3) and are therefore not considered relevant for attention. However, peptide selection and presentation undergoes a most elaborated extraction from the whole available proteome. It is known that HLA-F confers a beneficial effect on disease outcome during HIV-1 infections. The interaction with the NK cell receptor initiates an antiviral downstream immune response and lead to delayed disease progression. During the time of HIV infection, HLA-F expression is upregulated, while its interaction with KIR3DS1 is diminished. The non-polymorphic nature of HLA-F facilitates the conclusion that understanding HLA-F peptide selection and presentation is essential to a comprehensive understanding of this dynamic immune response. Utilizing soluble HLA technology we recovered stable pHLA-F*01:01, 01:03 and 01:04 complexes from K562 cells and analyzed the peptides presented. Utilizing a sophisticated LC-MS-method, we analyzed the complete *K562* proteome and matched the peptides presented by the respective HLA-F subtypes with detected proteins. All peptides featured a length of 8 to 24 amino acids and are not N-terminally anchored; the C-terminus is preferably anchored by Lys. To comprehend the alteration of the pHLA-F surface we structurally compared HLA-F variants bound to selected peptides. The peptides were selected from the same cellular content; however, no overlap between the proteomic source of F*01:01, 01:03 or 01:04 selected peptides could be observed. Recognizing the balance between HLA-F expression, HLA-F polymorphism and peptide selection will support to understand the role of HLA-F in viral pathogenesis.

## 1. Introduction

HLA-F belongs to the family of HLA-Ib molecules and is of the whole HLA genes the most conserved locus; the RNA expression is highly tissue specific, e.g., bone marrow, immune cells, lung, liver, gall bladder, gastrointestinal tract and skin (https://www.proteinatlas.org), while its protein expression is highly dependent on the cellular health status. One of the main and exquisite immune functions of HLA-F is the upregulation on the surface of HIV infected CD4^+^ T cells [1]. The presence of HLA-F enables natural killer (NK) cells to recognize the HIV infection and to initiate the cytolysis of the CD4^+^/HIV^+^/HLA-F^+^ T cells via binding of its cognate receptor KIR3DS1 [2]. This exclusive ligand-receptor interaction is diminished in the late phase of HIV infection [1], while the expression of HLA-F remains equal. It has been suggested that HLA-F is presented to the immune system as an open conformer (OC) molecule lacking peptide presentation and association to β2-microglobuline (β2m). Initially it was assumed that HLA-F is entirely unable to present peptides. Yet, it could be demonstrated recently that HLA-F is able to select, bind and present peptides [3,4]; trimeric complexes of HLA-F heavy chain, β2m and peptide could be isolated following their assembly within the cells. Those HLA-F bound peptides are of unusual length and the binding mode to the peptide binding region reveals an open end conformation without the classical pocket A or pocket B engagement; this observation explains the existence of HLA-F OCs. Structural and functional analysis illustrated that the engagement of HLA-F with its cognate NK cell receptor is dependent on the presence or absence of a bound peptide [3].

Peptide presentation is the main key to immune recognition [5]; the features of bound peptides determine the structure of an HLA molecule, and therefore, the recognition of its cognate receptor [6,7]. The amino acid composition of the HLA heavy chain dictates the mode of peptide selection [8,9], and therefore, the origin, the sequence and the binding affinity of a finally presented peptide. Analyzing HLA allele specific peptide repertoires (http://www.syfpeithi.de) [10] enabled to assign the importance of distinct amino acids within the HLA heavy chain [11] and to predict the impact of allelic mismatches. The variability of HLA variants is driven by evolutionary selection and pathogenic pressure [12].

HLA-Ib molecules play a role as part of immune evasion strategies and/or are mediators of immune tolerance and are therefore characterized by their restricted tissue distribution [13]. Despite their marginal polymorphic nature and proposed immunological invariability, most HLA-Ib allelic variants differ substantially in their peptide profiles [14,15,16]; this variability results in differential immune responses [17,18]. All investigations to elucidate the function of HLA-F concentrate on the most frequent allelic variant HLA-F*01:01 (Table 1) [3,4,19,20,21]. A recent study on the haplotype lineages of HLA-F describes the relation between haplotypes and RNA expression levels [22]; moreover, recently a significant association of HLA-F polymorphism on genomic level with chronic HBV infection has been suggested [23]. However, the functional impact of allelic mismatches on the protein and peptidome has not yet been comprehensively described.

To date, 30 alleles encoding for five proteins (HLA-F*01:01, F*01:02, F*01:03, F*01:04, F*01:05) have been described [26,27]. All residues polymorphic are located outside the PBR, and therefore not suggested to alter peptide features such as the binding motif [11]. To assign a significance to HLA-F polymorphism we analyzed the allelic variants HLA-F*01:01, F*01:03 and F*01:04. To ensure plausibility, we omitted the functional analysis of the allelic variants F*01:02 and F*01:05 since those variants differ from the F*01:01 by amino acid residues p.−9A>V and p.278P>L, respectively; those residues are located in the signal peptide and the transmembrane domain and are therefore not of functional impact for peptide binding [11].

Appreciating the balance between HLA-F polymorphism and peptidome diversity will establish a comprehension of the role of HLA-F in viral pathogenesis.

## 2. Results

### 2.1. HLA-F Restricted Peptides of All Allelic Variants Exhibite Non-Canonical Length

**Figure 1:**
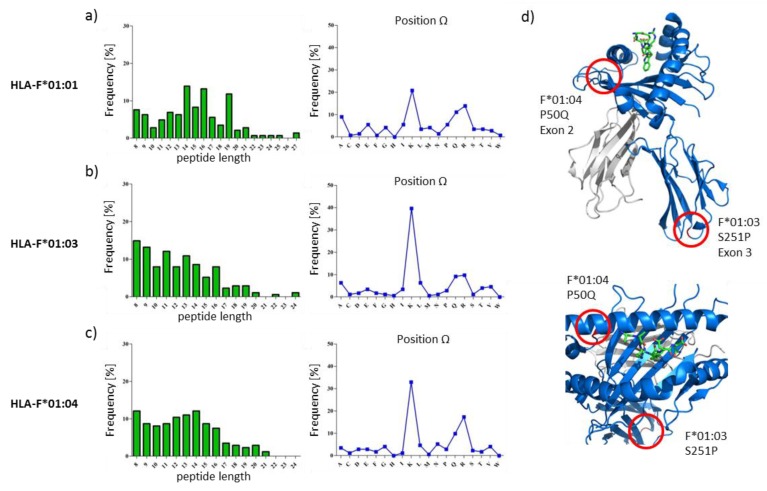
Features of HLA-F restricted peptides. (**a**–**c**) Length distribution and frequency of amino acid (AA) at position Ω of peptides presented by (**a**) HLA-F*01:01, (**b**) HLA-F*01:03 and (**c**) HLA-F*01:04. Peptide length is given on the *x*-axis, percentage of observed peptides on the *y*-axis. The respective AA is given on the *x*-axis, percentage of observed AA on the *y*-axis. (**d**) Structure of HLA-F*01:01 (5KNM) from Dulberger et al. [3]. Positions of AA exchanges between three allelic variants are circled in red. P50Q discriminates HLA-F*01:01 from F*01:04; S251P discriminates F*01:01 from 01:03.

**Figure 2:**
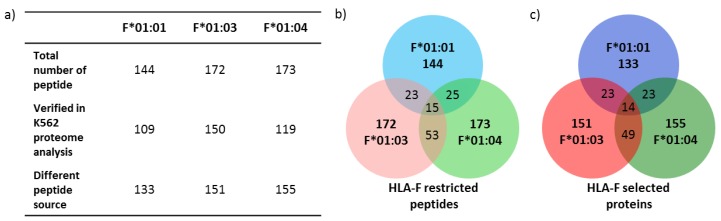
Features of HLA-F restricted peptides. (**a**) Overview of total number of HLA-F restricted peptides and the amount of different peptide source. (**b**) Venn diagram of HLA-F restricted peptides of three allelic variants. (**c**) Venn diagram of HLA-F selected proteins of HLA-F*01:01, HLA-F*01:03 and HLA-F*01:04.

The HLA class I deficient cell line *K562* were successfully transduced with vectors encoding for the soluble HLA-F variants (HLA-F*01:01, HLA-F*01:03, HLA-F*01:04). To ensure high peptide coverage over several time points, sHLA-F containing supernatant was harvested bi-weekly over a period of 2 months from bioreactors and following affinity chromatography of trimeric sHLA-F complexes, bound peptides were recovered and mass spectrometrically analyzed (Figure 1, Tables S1–S3). A total of 144, 172 and 173 peptides restricted to HLA-F*01:01, HLA-F*01:03 and HLA-F*01:04 were sequenced (Figure 2a, Tables S1–S3). The respective peptide sequences are given in Tables S1–S3. The HLA-F restricted peptides showed relatively similar length features. The majority of HLA-F restricted peptides exhibited non-canonical length for all allelic variants. The peptides presented by HLA-F*01:0x in *K562* cells were 8 to 27 AAs in length (Figure 1). Longer peptides (>10 AA) were observed in more than 83.5%, 64.4% and 71.2% for HLA-F*01:01, HLA-F*01:03 and HLA-F*01:04, respectively. In HLA-F*01:01 restricted peptides 14-mer (13.9%) and 16-mer (13.2%) were found most frequently. 8-mer (14.0%) and 9-mer (13.4%) peptides were found most frequently in HLA-F*01:03. In HLA-F*01:04 restricted peptides 8-mer (12.1%) and 14-mer (12.1%) were found most frequently.

### 2.2. The Peptide Binding Motif of HLA-F*01:0x Exhibits a High Frequency of Polar and Positively Charged AAs at pΩ

sHLA-F*01:0x restricted peptides derived from *K562* cells shared polar and positively charged AAs as pΩ anchor. Other anchor AAs could not be identified. Analysis of the anchoring position showed that all allotypes exhibited Lysin as the dominant C-terminal anchor. HLA-F*01:01 restricted peptides exhibited Lysin (20.8%) and Argenine (13.8%) as auxiliary anchor. HLA-F*01:03 and HLA-F*01:04 restricted peptides exhibited only Lysin (40.1% and 32.9%) as anchor.

### 2.3. Only 9.2% of Peptides Are Shared between the Three Allelic HLA-F Variants

Peptide overlaps between the analyzed peptide repertoires of the three allelic HLA-F variants showed a total of 9.2% of all peptides (Figure 2). Peptide sequences are given in Table 2. 10 of the 15 peptides shared between the three allelic variants exhibits non-canonical length (>10 AA). Between F*01:01 and F*01:03, 14.6% of overlapping peptides were found. An overlap of 15.7% peptides were found between F*01:01 and F*01:04. F*01:03 and F*01:04 shared 30.7% peptides. Although the three allelic variants shared the same peptide features, there are only a few overlaps in the peptide sequence.

### 2.4. Features of HLA-F Selected Proteins

**Figure 3:**
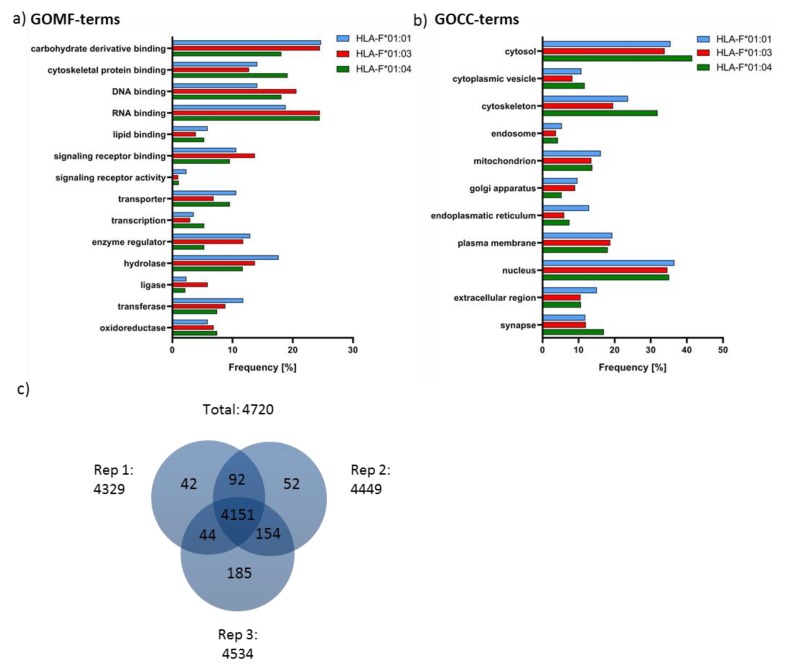
Data analysis of identified HLA-F selected proteins and proteome analysis of *K562* cells. (**a**) Gene ontology molecular function (GOMF) analysis of HLA-F presented peptides. Depicted are selected gene ontology GO terms and the involvement of the protein in every category of selected GOMF. (**b**) Gene ontology cellular component (GOCC) analysis of HLA-F presented peptides. Depicted are selected GO terms and the presence of the protein all cellular compartments. (**c**) Venn diagram of identified proteins within three biological replicates. Only proteins that were identified in all replicates were considered for quantification.

In order to verify the biological occurrence of the source protein of the HLA-F restricted peptides, proteome analysis of *K562* cells was performed (Figure 2a and Figure 3c). All known source proteins of the peptide could also be detected in the proteome analysis of *K562* cells. To understand the HLA-F peptide selection in the three allelic variants, we analyzed the protein source of HLA-F restricted peptides for their molecular function (Figure 3a) and occurrence in cellular components (Figure 3b). The comparison of the molecular function of source proteins of the three allelic variants showed that HLA-F*01:01 selected proteins are less involved in RNA binding (21.1%) than HLA-F*01:03 (29.8%) or HLA-F*01:04 (29.0%) selected proteins. HLA-F*01:01 selected proteins are more involved in enzyme regulator processes (18.8%) than HLA-F*01:03 (9.5%) or HLA-F*01:04 (7.2%) presented proteins. In consideration of the gene ontology cellular components (GOCC) terms (Figure 3b), there was no remarkable difference between the three allelic variant. The peptides presented by the HLA-F allelic variants are mainly derived from cytosol and nucleus.

## 3. Discussion

Several studies attempted to determine the impact of allelic variability within the HLA system on immune function. The analysis of allele specific peptide binding profiles, repertoires and biophysical features has been most beneficial to identify HLA hc residues altering i) the pHLA structure, ii) the mode of peptide selection and iii) the origin of peptides. Therefore, the meticulous analysis of allele specific peptides is the key for the functional understanding of HLA molecules.

The non-classical MHC-Ib molecule HLA-F was first described as HLA-5,4 by Geraghty et al. in 1989 [28]. To date, HLA-F is the most enigmatic HLA-Ib molecule and its function still appears to be ambiguous. Thus far, it has been found that HLA-F regulates immunity during viral infection [1,20,29], pregnancy and autoimmunity [30] through its interaction with certain KIR receptors. Furthermore, expression of HLA-F is associated with a negative overall survival in non-small cell lung cancer [31] and gliomas [32]. Yet, the function of HLA-F in cancer still remains unknown. Compared to the classical HLA-Ia molecules (HLA-A, -B and -C) that consistently function as reporter of the health status of a cell for the adaptive immune system, HLA-Ib molecules (HLA-E, -G and -F) exhibit a diverse range of functions in adaptive and innate immunity while they appear to be structurally invariable. While the highly polymorphic HLA-Ia molecules activate the immune system during pathogenic invasion, each HLA-Ib molecule is highly specialized in its expression profile and immune function. In contrast to HLA-Ia molecules that are targets of immune evasion strategies, HLA-Ib molecules are upregulated during pathogenic episodes and represent mediators for immune tolerance; hence, the balance between HLA-Ia absence and HLA-Ib presence dictates the outcome of pathogenic episodes. Expression of HLA-E on the cell surface avoids the recognition of HLA-Ia empty and viral infected cells by NK cells [33] through HLA-E-NKG2A interaction. The interaction between HLA-E is allele- [16] and peptide- [15,34] specific; therefore, the selection of NKG2A-ligated HLA-E-peptides is the key to pathogenic immune escape or immune survival. The same holds true for HLA-G function, a molecule that confers protection to the fetus from the maternal immune system during pregnancy [35]. The presence of HLA-G is associated with immune tolerance [14,36,37]. The apparent invariability of HLA-G molecules has disguised its exquisite immune function. It could be described recently that HLA-G not only selects peptides allele-specific [14], but also tissue-specific [38]. Consequently, HLA-E and G molecules play a pivotal role in mediating immune tolerance in favor of the host immune system or in favor of the pathogen [33,39]. The discovery of peptide specificity sheds light on the immunological paradox of differential immune responses triggered from innate receptor interaction with invariable immune molecules. To this end, the question of which functional significance the existence of HLA-F allelic variants represent, arises. The remarkable immune function of HLA-F is its expression on the cell surface of HIV infected CD4^+^ T cells [1]. The interaction between HLA-F and KIR3DS1 leads to a delayed disease progression in CD4^+^/HIV^+^ cells [20]. During course of infection the amount of HLA-F remains consistent though its interaction with the receptor occurs to be diminished [1]. Not only the role of HLA-F in tumors, but also its regulation during viral infection seems to be highly ambiguous. Since it could be demonstrated that HLA-F is able to select, bind and present peptides [3,4], we suggest that understanding peptide selection is the key to decode the biological function of HLA-F. All investigations concerning interaction between HLA-F and its receptors concentrate on the most frequent allelic variant HLA-F*01:01 as OC or peptide-bound molecule. It could be recently demonstrated that HLA-F represents biophysical properties that are comparable with HLA-Ia molecules by presenting a distinct set of peptides. Due to the fact that the peptide assignment for each HLA allelic variant is specific and each pHLA molecule displays a unique landscape to its cognate immune receptor, it becomes obvious how beneficial the analysis and understanding of HLA-F alleles and their corresponding peptides would be.

In the present study, we investigated the peptide repertoires of HLA-F*01:01, HLA-F*01:03 and HLA-F*01:04. These alleles are distinguished by polymorphism outside the peptide binding region (p.50P>Q, F*01:04 and p.251S>P, F*01:03) either at the beginning of the β-sheet floor of the groove or in a loop region of α3-helix. Those polymorphisms do not directly interact with peptide binding [11] or binding of known receptors [40] and are therefore not considered relevant for attention. However, the polymorphism of the non-classical HLA-E and HLA-G are not supposed to impact peptide selection and/or peptide presentation as well; yet, it could be demonstrated that an AA exchange in the outer loop region of HLA-E impact peptide presentation and immune function significantly [15,16]. Furthermore, the polymorphisms distinguishing HLA-G variants that have been considered so far as insignificant for peptide presentation and NK cell engagement seem to play an essential role in immune recognition [14]. In this study, it could be shown that the polymorphisms between the allelic variants of HLA-F do not influence peptide binding features of the molecules. HLA-F presented peptides with non-canonical length. We could identify polar and positively charged AA such as lysine and arginine to anchor the peptides C-terminally. These findings are slightly different from our previous study, where arginine exclusively could be identified as the dominant C-terminal AA [4]. In the present study arginine was the second most common C-terminal AA. This phenomenon could be explained by the source of proteins that are available from the cellular proteome since different cell lines have been used for HLA-F peptide fishing. We could recently demonstrate the significance of the proteomic source for peptide selection of invariant non-classical HLA-G molecules and the associated outcome [38]. An N-terminal anchor AA could, however, not be identified. These findings are reminiscent of a previous HLA-F analysis, where HLA-F*01:01 binds long peptides that protrude out of the PBR N-terminally [3,4]. The three allelic variants of HLA-F do not form a classical pHLA structure [4]. The binding of long peptides lead to the formation of a very flexible accessible surface of pHLA-F molecules for corresponding immune receptors. Several studies demonstrated that peptides are able to dictate the interaction partner of the HLA ligand; HLA-E bound to certain peptide constitutes either a ligand for the inhibitory receptor NKG2A/CD94 or activating receptor NKG2C/CD94 on NK cells [34]. However, in this study, the peptides presented by the HLA-F allelic variants share the same peptide features with little peptide sequence overlap between the three allelic variants (Figure 2). It has not been evaluated, yet, to what extend HLA-Ib molecules select peptides from the whole proteomic content. The little structural variability between the pHLA-F molecules raises the question of the establishment of HLA-F allelic variants [27]. Since polymorphism evolved over time to overcome pandemic episodes [41,42,43] or to support immune tolerance for reproduction [17], it seems obvious that the establishment of HLA-F alleles substantiates a certain function and feature biological differences. Therefore, we analyzed the protein source of peptides presented by HLA-F (Figure 2c); there was almost no overlap between the peptide source proteins. The gene ontology (GO) analysis revealed that the proteins of the three allelic variants derived from the same cellular compartment, but they exhibit different molecular function (Figure 3). We detected the most interesting fact that HLA-F*01:03 presented proteins that are more involved in DNA binding than the other two allelic variants. It was reported that HLA-F*01:03 is associated with a decrease level of the HBV DNA. Comparing this observation with the analysis of peptide source proteins, it becomes obvious that not only the peptide itself, but also the origin of the peptide will contribute to a better understanding of HLA-F and its unexplored function in immunity. The data presented in this study showed that not only the polymorphism but also the proteomic source plays an essential role in the peptide presentation.

## 4. Material and Methods

### 4.1. Maintenance of Cell Lines

All cell lines were cultured at 37 °C and 5% CO_2_. The recombinant HLA class I negative *K562* cell lines expressing sHLA-F*01:0x molecules (soluble, exon 1-4) were cultured in RPMI 1640 (Lonza, Basel, Switzerland) supplemented with 10% heat inactivated fetal calf serum (FCS, Lonza), 2 mM L-glutamine (c. c. pro, Oberdorla, Germany), 100 U/mL penicillin and 100 µg/mL streptomycin (c. c. pro).

The human embryonal kidney cell line *HEK293T* (ATCC, Manassas, VA, USA ) was maintained in Dulbecco modified eagle medium (DMEM , Lonza) supplemented with 10% heat inactivated FCS, 2 mM L-glutamine, 100 U/mL penicillin, 100 µg/mL streptomycin and 1 mg/mL Geneticin^®^ (Life Technologies, Carlsbad, CA, USA).

### 4.2. Cloning of HLA-F Encoding Contructs

Constructs encoding for soluble HLA-F (exon 1-4) were generated from *HEK293T* cDNA via PCR. The sequence for soluble HLA-F*01:01 along with an N-terminal V5-His6 tag was cloned into the lentiviral vector *pRRL.PPT.SFFV.mcs.pre* as previously described [4]. To generate sHLA-F*01:03 and sHLA-F*01:04 encoding constructs, site-direct mutagenesis was used. Constructs for sHLA-F*01:03 were generated by introducing a single point mutation at position c.814T>C and at position c.212C>A for generating sHLA-F*01:04. The constructs were verified through genomic sequencing.

### 4.3. Stable Transduction of K562 Cells with Lentivirus Encoding for HLA-F*01:0x Molecules

Soluble HLA-F molecules (sHLA-F) were expressed in HLA class I negative *K562* cells according to the method described by Ho et al. [4].

Lentiviral particles were produced in *HEK293T* cells. *HEK293T* cells were transfected with the target plasmid for sHLA-F*01:0x (10 µg/5×10^6^ cells) along with the packaging and envelope vectors *psPAX2* and *pmD2G* (each 5 µg/5×10^6^ cells). *K562* cells were stably transduced with lentiviral particles encoding for the different HLA-F variants. The expression of trimeric sHLA-F*01:0x molecules was confirmed by HLA class I-specific ELISA as previously described [44].

### 4.4. Large-scale Production of sHLA-F*01:0x Molecules

Utilizing soluble HLA technology, *CeLLine* bioreactors (Integra Biosciences, Biebertal, Germany) were used for large scale production of recombinant sHLA-F*01:0x molecules [45]. Cell culture supernatant containing sHLA-F*01:0x molecules were harvested weekly, centrifuged and filtered through a 0.45 µM membrane (Millipore, Schwalbach, Germany) to remove cells and debris. Trimeric sHLA-F*01:0x molecules were purified using an N-hydroxy-succinimide (NHS)-activated *HiTrap* column (Life Technologies) coupled to the mAb W6/32. Purified proteins were verified quantitatively via an HLA class I-specific ELISA and qualitatively via SDS gel electrophoresis (Figure S1) and Western blot analysis Figure S2).

### 4.5. LC-MS Analysis of sHLA-F*01:0x Restricted Peptides and the Proteome

To elute peptides from purified sHLA-F*01:0x complexes, trifluoric acid (TFA, J. T. Baker, Phillipsburg, NJ, USA) was added in a final concentration of 0.1%. Peptides were separated from the heavy chain and light chain by using an Amicon Ultra centrifugal tube (Millipore, Schwalbach, Germany) with a 10 kDa cut-off membrane. Peptides were further purified using ZipTips (0.6 µl C18 resin, Merck, Darmstadt, Germany) and 50% Acetonitrile (ACN)/0.1% TFA for elution. Extracted peptides were dried via vacuum centrifugation in a Speedvac (Thermo Fischer, Rockford, IL, USA) and resolved in 2% ACN/0.1% TFA for LC/MS analysis.

For proteome analysis of *K562* cells, 5×10^6^ cells were lysed using 500µL RIPA buffer; the cell suspension was thoroughly vortexed and incubated on ice for 30 min and frozen at −80 °C. Cells were thawed on ice and homogenized by 3× 30 s bursts of ultra-sonication. Following centrifugation of lysates (15 min, 13,000 rpm, 4 °C), protein containing supernatant was harvested and the protein concentration was ascertained using the Bicinchoninic acid assay (BCA) Protein Quantitation Kit (Interchim, San Diego, CA, USA). 50 µg of protein was heated at 95 °C for 5 min, alkylated by adding 1 µL 40% acrylamide at RT and separated by SDS gel electrophoresis. Gels were stained with Coomassi SimplyBlue™ SafeStain (Thermo Fischer). To reduce complexity and thereby increase the amount of identified proteins, samples were fractionated. Each lane was sliced into six fractions that were manually cut into small cubes. Gel pieces were destained with 50% ACN/50mM Ammoniumcarbonat (ABC) and dehydrated with 100% ACN and dried via vacuum centrifugation. After rehydration in 10 ng/μL trypsin, 10% ACN/20mM ABC, samples digestion was performed with trypsin o/n at 37 °C and 350 rpm. Protein digestion was stopped by adding 50% ACN/0.5% TFA. After an additional dehydration step, dried peptides were solved in 30 µL 2% ACN/0.1% TFA for LC/MS analysis.

The LC/MS analysis was performed using a Dionex Ultimate 3000 high-performance LC system and a LTQ Orbitrap Lumos Mass Spectrometer (Thermo Fisher Scientific) in data-dependent acquisition (DDA) mode. Samples were separated by reverse phase chromatograph and ionized via electro-spray ionization with an emitter voltage of 1.35 kV. Orbitrap mass analyzer recorded the survey scans; the most intense precursors with a charge of 2 or higher were chosen for collision induced dissociation (CID) fragmentation. Normalized collision energy of 38% for 10 ms was used. MS/MS spectra were acquired in the ion trap of the mass spectrometer.

Proteome data were analyzed using MaxQuant software (Version 1.6.50, https://www.maxquant.org/) and the human entries of Uniprot data base (https://www.uniprot.org/). Proteins were stated identified if false discovery rate (FDR) on protein and peptide level was less than 0.01. For identification of HLA-enriched peptides, MS/MS spectra were analyzed using proteome discoverer software (Version 1.4, Thermo Fisher Scientific) and the human entries of Uniprot data base. Peptides were stated identified if they had a minimum length of eight peptides and an FDR of less than 0.01.

## Figures and Tables

**Table 1:** Identification methods of interaction between HLA-F and its cognate NK cell receptors.

Type of HLA-F	Allelic Variant	NK Cell Receptor	Method of Identification	Reference
pHLA-F tetramer	F*01:01	ILT-2	SPR	[24]
pHLA-F tetramer	F*01:01	ILT-4	SPR	[24]
HLA-F OC	F*01:01	KIR3DL2	rKIR-Fc binding to HLA-I coated beads; SPR; rKIRζ jurkat reporter cell assay	[1,3,25]
HLA-F OC	F*01:01	KIR2DS4	Pull-down precipitation; SPR	[25]
HLA-F OC	F*01:01	KIR3DL1	SPR; rKIR-Fc binding to HLA-I coated beads	[1,2]
HLA-F OC	F*01:01	KIR3DS1	pull-down precipitation; rKIR-Fc binding to HLA-I coated beads; SPR; rKIRζ jurkat reporter cell assay	[1,2,3]
pHLA-F	F*01:01	ILT-2	Biolayer interferometry assay; X-ray crystallography	[3]

pHLA-F tetramers were used to identify NK cell receptor ILT-2 and ILT-4. HLA-F OC is a ligand for KIR3DL2, KIR2DS4, KIR3DS1. pHLA-F could be confirmed to be the ligand of ILT-2. pHLA-F = peptide bound HLA-F; HLA-F OC = open conformer of HLA-F; rKIR = recombinant KIR; SPR = surface plasmon resonance.

**Table 2:** Shared peptides between HLA-F*01:01, F*01:03 and F*01:04.

Sequence	Length	Source
KVGDDIAK	8	60S ribosomal protein L12
MAHMASKE	8	Glyceraldehyde-3-phosphate dehydrogenase
APNHAVVTR	9	Serotransferrin
AVTKYTSAK	9	Histone H2B type 1-K
AGFAGDDAPR	10	Actin, cytoplasmic 1
AGEKVEKPDTK	11	60S ribosomal protein L6
EITALAPSTMK	11	Actin, cytoplasmic 1
IVTDRETGSSK	11	Nucleolin
MYLGYEYVTAIR	12	Serotransferrin
TVLIMELINNVAK	13	ATP synthase subunit beta, mitochondrial
VNVDEVGGEALGR	13	Hemoglobin subunit beta
VTGYNDPETGNII	13	Desmoplakin
SYELPDGQVITIGNER	16	Actin, cytoplasmic 1
TGAIVDVPVGEELLGR	16	ATP synthase subunit alpha, mitochondrial
TITLEVEPSDTIENVK	16	Ubiquitin-40S ribosomal protein S27a

## Supplementary Materials

Supplementary materials can be found at https://www.mdpi.com/1422-0067/20/22/5572/s1.
