# Supplementary material for: HLA-F Allele-Specific Peptide Restriction Represents an Exceptional Proteomic Footprint
Source: Int J Mol Sci. 2019 Nov 8;20(22):5572. doi: 10.3390/ijms20225572 (PMC6888383; doi:10.3390/ijms20225572)
Supplement: Supplementary file 1 [file ijms-20-05572-s001.pdf]

Supplementary Materials:

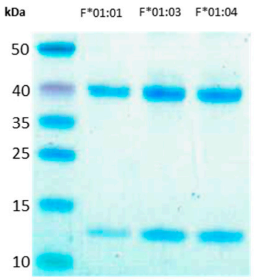

Figure S1. SDS-PAGE of purified sHLA-F allelic variants. SDS-PAGE of sHLA-F allelic variants after affinity chromatography; all samples were diluted 1:2; Spectra Multicolor Broad Range was used as maker; HLA-F hc/V5-His6 (~39 kDa) and β2m (11.7 kDa).

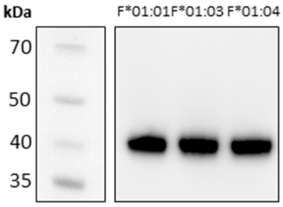

Figure S2. Western Blot analysis of purified HLA-F allelic varfiants. Western Blot of sHLA-F allelic variants after affinity chromatography; Spectra Multicolor Broad Range was used as maker; anti HLA-F (3D11) antibody was used for the detection of HLA-F heavy chain.

Table S1. HLA-F*01:01 restricted peptides[a].

Sequence	Length	Source	Accession Number
AGGQLTKL	8	Poly(rC)-binding protein 2	H3BRU6
KNVALINQ	8	Inositol 1,4,5-trisphosphate receptor type 1	Q14643
KVGDDIAK	8	60S ribosomal protein L12	P30050
KVTQDELK	8	Nucleolin	P19338
LEKGLDGA	8	Isoform 2 of Dermcidin	P81605-2
MAHMASKE	8	Glyceraldehyde-3-phosphate dehydrogenase	P04406
QEELQQLR	8	Plectin	Q15149
RGAARLVG	8	Helicase SKI2W	Q15477
RSGSRVAV	8	Mediator of RNA polymerase II trans	M0R064
VDIINAKQ	8	Triosephosphate isomerase	P60174
VGSRGSLR	8	Leucine-rich repeat flightless-interacting protein 1	Q32MZ4
APNHAVVTR	9	Serotransferrin	P02787
AVTKYTSAK	9	Histone H2B type 1-K	O60814
AVTKYTSSK	9	Histone H2B type 1-C/E/F/G/I	P62807
GAGGLLSVK	9	Protein KRBA1	A5PL33
KAGGAQLGV	9	Collagen alpha-1(II) chain	P02458
LQAGATLAG	9	Isoform 3 of Ubiquitin-conjugating enzyme E2 L3	P68036-3
		Zinc finger protein 407	Q9C0G0
		Cadherin-related family member 1	Q96JP9
LSSPLASGA	9	Cytochrome b-c1 complex subunit 1, mitochondrial	P31930
QGRVNQLVQ	9	Isoform 2 of Rho GTPase-activating protein 4	P98171-2
RVEAKPEVQ	9	Ras GTPase-activating protein-binding protein 2	Q9UN86
AGFAGDDAPR	10	Actin, cytoplasmic 1	P60709
AIGILNNSAS	10	Isoform 2 of NF-kappa-B-repressing factor	O15226-2
IAGQVAAANK	10	40S ribosomal protein S19	P39019

Sequence	Length	Source	Accession Number
LKAENNSEVG	10	ATP-dependent RNA helicase A	Q08211
AGEKVEKPDTK	11	60S ribosomal protein L6	Q02878
DAVEDLESVGK	11	Isoform 2 of Dermcidin	P81605-2
EITALAPSTMK	11	Actin, cytoplasmic 1	P60709
IVTDRETGSSK	11	Nucleolin	P19338
NNLEALEDFEK	11	Lipocalin-1	P31025
PEPAKSAPAPK	11	Histone H2B type 1-K	O60814
SAPGGGSKVPQ	11	Nucleophosmin	P06748
ATGQKAAPAPKA	12	60S ribosomal protein L14	P50914
DSLIQCPIDTRK	12	Actin-related protein 10	Q9NZ32
EESFQEQSALAA	12	tRNA-splicing endonuclease subunit Sen34	Q9BSV6
KQSSQIQNSACI	12	Tripartite motif-containing protein 65	Q6PJ69
MYLGYEYVTAIR	12	Serotransferrin	P02787
QKENAGEDPGLA	12	Isoform 2 of Dermcidin	P81605-2
SSGGETLWNLPA	12	Protein dispatched homolog 1	Q96F81
		DNA polymerase subunit gamma-2, mitochondrial	Q9UHN1
STDYGIFQINSR	12	Lysozyme C	P61626
VEFHPTDANTII	12	Echinoderm microtubule-associated protein-like 4	Q9HC35
		Tyrosine-protein kinase receptor	A6P4V4
YFPTQALNFAFK	12	ADP/ATP translocase 4	Q9H0C2
EETAATASAIEAM	13	TRIO and F-actin-binding protein	Q9H2D6
EVGMKYRNLILKP	13	Neurolysin, mitochondrial	Q9BYT8
LELQCLEDGGPGP	13	Transmembrane protein 134	Q9H6X4
LSSNLAQRRGMKR	13	Ribosomal protein S6 kinase	F2Z2J1
SPAAGSSPGKPPR	13	Cystatin-C	P01034
SRGSYQLQAQMNR	13	WD repeat-containing protein 13	Q9H1Z4
TVLIMELINNVAK	13	ATP synthase subunit beta, mitochondrial	F8W079
VNVDEVGGEALGR	13	Hemoglobin subunit beta	P68871
VTGYNDPETGNII	13	Desmoplakin	P15924
DLKVLDMARAGFLL	14	Solute carrier family 13 member 2	Q13183
DSYLKTRSPVTFLS	14	CCR4-NOT transcription complex subunit 1	A5YKK6
EDPQTFYYAVAVVK	14	Serotransferrin	P02787
ENPNNFQEVAADSS	14	Pancreatic triacylglycerol lipase	P16233
EVLIDALVDGQVVA	14	Beta-catenin-like protein 1	Q8WYA6
FDEFFSEGCAPGSK	14	Serotransferrin	P02787
GAAKEAAGKSSGPT	14	Heterogeneous nuclear ribonucleoprotein U	Q00839
GCNHKLELALSMIK	14	Isoform 13 of Myomegalin	Q5VU43-13
GVDEVTIVNILTNR	14	Isoform 2 of Annexin A2	P07355-2
KDATSKVSALLGRI	14	ATP synthase subunit beta, mitochondrial	F8W079
KEIVLMLCQAFGII	14	Taste receptor type 2 member 20	P59543
KVEQAVETEPEPEL	14	Apolipoprotein E	P02649
LGANSLLDLVVFGR	14	Succinate dehydrogenase [ubiquinone] flavoprotein subunit, mitochondrial	P31040
LISQIVSSITASLR	14	Tubulin alpha chain	F5H5D3
LRSDNVVEGNCADE	14	Glutamyl-tRNA(Gln) amidotransferase subunit C, mitochondrial	O43716
MNTQILLMAVKNNS	14	Mutant methylmalonyl CoA mutase	A0A0G3IDQ3
NMVIMLIGNKSDLE	14	Ras-related protein Rab-2B	Q8WUD1
PMALSENSGMNPIQ	14	T-complex protein 1 subunit epsilon	P48643
SQGGSTLGMSSRHN	14	Cysteine/serine-rich nuclear protein 3	Q8WYN3
SSDMQSVLSCNTTL	14	Inactive dual specificity phosphatase 27	Q5VZP5
AVFVDLEPTVIDEVR	15	Tubulin alpha chain	F5H5D3
GEDIDTISPTLGFNI	15	ADP-ribosylation factor-like protein 2	P36404
GILMGVPVPFPIPEP	15	NPC intracellular cholesterol transporter 2	P61916
KLQVLDLQDVDENFW	15	PRAME family member 1	O95521
KPELQTSASQQMLNF	15	Isoform 3 of SWI/SNF complex subunit SMARCC2	Q8TAQ2-3
KPNGNFLNKDFLELR	15	RNA-binding protein 44	Q6ZP01
PEKDLHEGFHYIIHE	15	Serpin A12	Q8IW75
QSLPLRVVCQPAGLP	15	Ubiquitin carboxyl-terminal hydrolase 34	Q70CQ2

Sequence	Length	Source	Accession Number
SLGVILFICLSGYPP	15	Serine/threonine-protein kinase Chk2	O96017
SSKGDQMISVNVCIK	15	Germinal-center associated nuclear protein	O60318
TSSLLFCLANSNYER	15	NADH-ubiquinone oxidoreductase chain 4	P03905
TVLMKDAIKPNLMQT	15	Tubulin alpha chain	F5H2F4
AFPSQGTKRPGGPSNV	16	Vacuolar protein sorting-associated protein 53 homolog	Q5VIR6
ANQEIAAMIDTEFAKQ	16	TGF-beta-activated kinase 1 and MAP3K7-binding protein 1	Q15750
AVAEPQIAMFCGKLNM	16	Amyloid-like protein 2	Q06481
AVSGSPGAAITPLPST	16	Crk-like protein	P46109
CASVTGQSIANTIVLM	16	Integrin alpha-8	P53708
		Voltage-dependent P/Q-type calcium channel subunit alpha-1A	O00555
FAPNILENSEALELVK	16	Gamma-enolase	P09104
GALAAYTQALGLDATP	16	Protein unc-45 homolog A	Q9H3U1
GLLGVNGAGKTTIFKM	16	ATP-binding cassette sub-family A member 12	Q86UK0
GLSTQGQAFPAQQLLK	16	Transcription factor SPT20 homolog-like 1	Q3ZLR7
GSGSSEGFDPPATDRQ	16	Y-box-binding protein 3	P16989
KTNKSMYECKKSDQYD	16	MAGUK p55 subfamily member 7	Q5T2T1
PADAGAFNAPVINRFT	16	Y-box-binding protein 3	P16989
PSNPAALPVASDSSPM	16	Retinoic acid-induced protein 1	Q7Z5J4
RGEVAPDAKSEFFQQA	16	Galectin-1	F8WEI7
SSPCDSNSSSLPRGDV	16	PWWP domain-containing DNA repair factor 3A	Q2TAK8
SYELPDGQVITIGNER	16	Actin, cytoplasmic 1	P60709
TGAIVDVPVGEELLGR	16	ATP synthase subunit alpha, mitochondrial	P25705
TITLEVEPSDTIENVK	16	Ubiquitin-40S ribosomal protein S27a	P62979
VSPTHDPQVAVDALLQ	16	E3 ubiquitin-protein ligase SH3RF3	Q8TEJ3
		Protein cramped-like	Q96RY5
ALLQNLYQPTGGQLLLD	17	Antigen peptide transporter 1	Q03518
CVGDSGGPLMCDGVLQG	17	Kallikrein	P06870
GLVQALGAHLYQNVFAC	17	Tubulin alpha chain	F5H2F4
GTNGSNATPSENTSPTA	17	Ubiquilin-1	Q9UMX0
NTTLGATQLSERPACVK	17	Phosphatidylinositol 4-kinase alpha	P42356
PCSKRYLWQTIMKEVRE	17	ATP-binding cassette sub-family A member 13	Q86UQ4
QVKMCLNPHCLALHSFI	17	HMG domain-containing protein 3	Q12766
SSNPIIPSQSAASSAIA	17	Corneodesmosin	G8JLG2
IESQIQTSRNLDPQPPIE	18	Transketolase-like protein 1	P51854
INTPLTTTSGNLHGQPVS	18	Isoform 2 of Arginase-1	P05089-2
NGARAEVSQFAACNLAQI	18	Melanotransferrin	P08582
PGRAPAGAPRPPAEAGAA	18	Maestro heat-like repeat-containing protein family member 6	E9PPP7
		Telomerase reverse transcriptase	O94807
VPAEACRRRAACVLFTVM	18	BAI1-associated protein 3	O94812
AIAELGIYPAVDPLDSTSR	19	ATP synthase subunit beta, mitochondrial	P06576
ASSAGVLSTVQSAAQAVLQ	19	E3 ubiquitin-protein ligase HERC2	O95714
CKNKLFCVAELQLATTVSQ	19	Integrin alpha-E	P38570
		Vascular endothelial growth factor C	P49767
FFESFGDLSTPDAVMGNPK	19	Hemoglobin subunit beta	P68871
GATVGLGIQMCEGWRVEGQ	19	Septin-1	J3KNL2
		Thyroglobulin	P01266
IDGNCTPQNPPQKKKSPVG	19	Inactive histone-lysine N-methyltransferase 2E	Q8IZD2
LAVSYMSQVLEKEMKAQEQ	19	Mannose-1-phosphate guanyltransferase beta	Q9Y5P6
LMTSKTEVMLLNPYRVEEA	19	PMS1 protein homolog 1	P54277
LQAALGLGRAGWHWPAGRA	19	Probable cysteine--tRNA ligase, mitochondrial	Q9HA77
MQPPSLHAITSQQQLIQMK	19	Mediator of RNA polymerase II transcription subunit 12-like protein	Q86YW9
NFEMLQQHTVLQGQRLIAP	19	Mismatch repair endonuclease PMS2	P54278
NGLTGAKGAAGLPGVAGAP	19	Collagen alpha-2(I) chain	P08123
QAVCFLGFIIQLNWKKACQ	19	Multidrug and toxin extrusion protein 1	Q96FL8

Sequence	Length	Source	Accession Number
QREMKKKTVCTLNMGDKKY	19	Protein SCAF11	F8W6K1
QSLVSGGNQMCKEQKATKK	19	Uncharacterized protein KIAA0408	Q6ZU52
VAVLGASGGIGQPLSLLLK	19	Malate dehydrogenase, mitochondrial	P40926
YLDSSVRNSKSLKSDFYRY	19	Muskelin	Q9UL63
MEQKAKQNQVASPQPPHPGE	20	Isoform 2 of SURP and G-patch domain-containing protein 1	Q8IWZ8
PENPKIKLDGKLDQEGDDVQ	20	Leucine-rich repeat flightless-interacting protein 1	Q32MZ4
TRYNPEQTKVLSASQAFAAQ	20	1-acyl-sn-glycerol-3-phosphate acyltransferase epsilon	Q9NUQ2
DLYANTVLSGGTTMYPGIADR	21	Actin, cytoplasmic 1	P60709
FTESTTSDASEHASQSVPMVT	21	Nucleoprotein TPR	P12270
IMNGEADAMSLDGGFVYIAGK	21	Serotransferrin	P02787
TVAACSPFVLQFLQGRTLTLG	21	Baculoviral IAP repeat-containing protein 1	A0A0G2JS53
EGVYRKAVVLQAQNQMSEAHKL	22	Anaphase-promoting complex subunit 5	Q9UJX4
ASVQPLATQCFQLSNMFNPQTEE	23	RNA-binding protein 39	Q14498
VETGVLKPGMVVTFAPVNVTTEVK	24	Elongation factor 1-alpha 1	P68104
KLQPGSVKKVNESSLNWPQLENIGN	25	Calponin-3	Q15417
LEKHGIILNSEIATNGETSDTLNNVGY	27	Leucine-rich repeat flightless-interacting protein 1	Q32MZ4
NDANPETHAFVTSPEIVTALAIAGTLK	27	Aconitate hydratase, mitochondrial	A2A274

[a] Peptides are presented in N-terminal to C-terminal orientation.

Table S2. HLA-F*01:03 restricted peptides[a].

Sequence	Length	Source	Accession Number
VLSSIEQK	8	14-3-3 protein gamma	P61981
KVGDDIAK	8	60S ribosomal protein L12	P30050
IVDVKANK	8	60S ribosomal protein L23a	A8MUS3
AGVKINPK	8	Glutamate dehydrogenase 1, mitochondrial	P00367
MAHMASKE	8	Glyceraldehyde-3-phosphate dehydrogenase	P04406
KALPPEKK	8	Heterogeneous nuclear ribonucleoprotein U	Q00839
AAQEEYVK	8	Isoform 2 of Fructose-bisphosphate aldolase A	P04075-2
VGDKIATR	8	Isoform 2 of Transketolase	P29401-2
NQELRQVK	8	Isoform 3 of Importin-5	O00410-3
FNRAAPGA	8	Non-POU domain-containing octamer-binding protein	Q15233
AAKVVPVK	8	Nucleolin	P19338
SKEYFSKQ	8	Peroxiredoxin-1	Q06830
RSENVLSR	8	PH and SEC7 domain-containing protein 2	Q9BQI7
ALASRTQQ	8	Protein FAM188B	Q4G0A6
VEAKPEVQ	8	Ras GTPase-activating protein-binding protein 2	Q9UN86
SPAVPVKK	8	Serine/arginine repetitive matrix protein 1	A9Z1X7
AQVRIGGK	8	Transcription factor BTF3	P20290
VDIINAKQ	8	Triosephosphate isomerase	P60174
KVVASKAQ	8	Endogenous Bornavirus-like nucleoprotein 1	P0CF75
AIQGAIEK	8	Acetyl-CoA acetyltransferase, mitochondrial	P24752
KGLGAQKV	8	ADP-ribosylation factor GTPase-activating protein 2	Q8N6H7
ALGAQIEK	8	Replication stress response regulator SDE2	Q6IQ49
RGAGQAKQ	8	DnaJ homolog subfamily A member 3, mitochondrial	Q96EY1
KQEIVAEK	8	Coronin-1C	Q9ULV4
NTGQRAVLK	9	40S ribosomal protein SA	P08865
AALLKASPK	9	60S ribosomal protein L14	P50914
AADIDQEVK	9	Cullin-associated NEDD8-dissociated protein 1	Q86VP6
SGTSEFLNK	9	Endoplasmin	P14625
VNASASSLK	9	Fascin	Q16658
KGGVASGFK	9	Gelsolin	P06396
AANMHAQIK	9	Growth/differentiation factor 15	Q99988
AANMHAQIK	9	Growth/differentiation factor 15	Q99988
SLIGKKGQQ	9	Histone H2A.Z	P0C0S5

Sequence	Length	Source	Accession Number
AVTKYTSSK	9	Histone H2B type 1-D	P58876
AVTKYTSAK	9	Histone H2B type 1-K	O60814
AVELAANTK	9	Isoform 2 of Transketolase	P29401-2
STAAQQELR	9	Myosin-9	P35579
QRAMLMRQQ	9	Nuclear receptor coactivator 1	Q15788
ISKEQGNVK	9	Phenylalanine--tRNA ligase beta subunit	Q9NSD9
GKEDALVTK	9	rRNA 2'-O-methyltransferase fibrillarin	P22087
APNHAVVTR	9	Serotransferrin	P02787
NADELVKQK	9	T-complex protein 1 subunit alpha	P17987
TATQLAVNK	9	T-complex protein 1 subunit eta	Q99832
VDENGKISR	9	Ubiquitin-40S ribosomal protein S27a	P62979
AGKSQQGAK	9	Zinc finger protein 750	Q32MQ0
QIVANAKGA	9	Perilipin	Q6FHZ7
RVSRSLDGA	9	Band 4.1-like protein 2	O43491
RATVVESSEK	10	14-3-3 protein gamma	P61981
TLVTRTQGTK	10	40S ribosomal protein S3a	P61247
NLQTVNVDEN	10	60S ribosomal protein L31	P62899
GYSFTTTAER	10	Actin, cytoplasmic 1	P60709
AGFAGDDAPR	10	Actin, cytoplasmic 1	P60709
SLTTPACLPL	10	DEP domain-containing protein 5	O75140
AASGEAKPKV	10	Histone H1.2	P16403
SLIGKKGQQK	10	Histone H2A.Z	P0C0S5
NTRETAQAIK	10	Isoform 3 of 60S ribosomal protein L17	P18621-3
AATSEGVQVK	10	Methyl-CpG-binding protein 2	A0A087WXF0
AAKVVPVKAK	10	Nucleolin	P19338
DINTDGAVNF	10	Protein S100-A8	P05109
LSGVSSNIQK	10	Rho GTPase-activating protein 30	Q7Z6I6
VADISGDTQK	10	Staphylococcal nuclease domain-containing protein 1	Q7KZF4
ANVGAGKKPKE	11	40S ribosomal protein S24	P62847
VLKAQAQSQKA	11	60S ribosomal protein L34	P49207
VTGGAASKLSK	11	60S ribosomal protein L35	P42766
AGEKVEKPDTK	11	60S ribosomal protein L6	Q02878
NFGIGQDIQPK	11	60S ribosomal protein L7a	P62424
HQGVMVGMGQK	11	Actin, cytoplasmic 1	P60709
EITALAPSTMK	11	Actin, cytoplasmic 1	P60709
AAGPPISEGKY	11	Corneodesmosin	G8JLG2
DAGAGIALNDH	11	Glyceraldehyde-3-phosphate dehydrogenase	P04406
VLMTQQPRPVL	11	H/ACA ribonucleoprotein complex subunit 3	Q9NPE3
AASGEAKPKVK	11	Histone H1.2	P16403
QVHPDTGISSK	11	Histone H2B type 1-D	P58876
PEPTKSAPAPK	11	Histone H2B type 1-D	P58876
PDPAKSAPAPK	11	Isoform 2 of Histone H2B type 2-F	Q5QNW6-2
SSQPLASKQEK	11	Isoform HMG-R of High mobility group protein HMG-I/HMG-Y	P17096-3
LPAITILGMAS	11	Mannose-1-phosphate guanyltransferase alpha	A0A0U1RRC2
AAEIDEEPVSK	11	Nascent polypeptide-associated complex subunit alpha, muscle-specific form	E9PAV3
VQKVQSVSQNK	11	Nucleolar protein 7	Q9UMY1
IVTDRETGSSK	11	Nucleolin	P19338
ATAVMPDGQFK	11	Peroxiredoxin-1	Q06830
ALVLVAMTLGQ	11	Transmembrane emp24 domain-containing protein 2	Q15363
TGETSRALSSSK	12	Activated RNA polymerase II transcriptional coactivator p15	P53999
AQNVGTTHDLLD	12	Bleomycin hydrolase	Q13867
SISGPGVDKEPF	12	Desmocollin-1	Q08554
VEFHPTDANTII	12	Echinoderm microtubule-associated protein-like 4	B5MBZ0
AGGSAALSPSKK	12	Histone H1x	Q92522
HAVSEGTKAVTK	12	Histone H2B type 1-D	P58876
TVTAMDVVYALK	12	Histone H4	P62805

Sequence	Length	Source	Accession Number
STDYGIFQINSR	12	Lysozyme C	P61626
SIRDTPAKNAQK	12	Nucleophosmin	P06748
MYLGYEYVTAIR	12	Serotransferrin	P02787
CRTSSGMRSQWP	12	Isoform 3 of Multiple coagulation factor deficiency protein 2	Q8NI22-3
QQLSKSQVEDPL	12	Mitogen-activated protein kinase 7	Q13164
LQIGTYANIAMV	12	5'-AMP-activated protein kinase subunit gamma-1	P54619
MIRTGEPGAGAS	12	Retinoblastoma-like protein 2	Q08999
		Isoform 2 of Kyphoscoliosis peptidase	Q8NBH2-2
GNPTVEVDLFTSK	13	Alpha-enolase	P06733
TVLIMELINNVAK	13	ATP synthase subunit beta, mitochondrial	P06576
LLTCSLNDSATEQ	13	Basigin	A0A087X215
NTCLSSNDISCLK	13	DEP domain-containing protein 4 (Fragment)	E9PGM3
KPVDPDGPENGPP	13	Desmocollin-1	Q08554
VTGYNDPETGNII	13	Desmoplakin	P15924
VNVDEVGGEALGR	13	Hemoglobin subunit beta	P68871
ALGQNPTQAEVLR	13	Myosin light chain 3	P08590
SGQNAWLFYHLAQ	13	Protocadherin alpha-C2	Q9Y5I4
LQEKNPAFKPVLA	13	Monofunctional C1-tetrahydrofolate synthase, mitochondrial	Q6UB35
HKSTRKVYAMKLL	13	Rho-associated protein kinase 1	Q13464
TVWPDHGVPETTQ	13	Receptor-type tyrosine-protein phosphatase beta	P23467
LFEEFANYEHVGI	13	Sodium/hydrogen exchanger 1	P19634
DFGVADLLPPDDK	13	Receptor tyrosine-protein kinase erbB-3	P21860
PNPTVAKTSPPVF	13	Transient receptor potential cation channel subfamily V member 3	Q8NET8
GTVQVNFYGDHTK	13	Serine/threonine-protein kinase PLK3	Q9H4B4
YGGVCKEDGDGLK	13	Tomoregulin-1	Q8IYR6
GSFPINGLHSHSE	13	Synaptotagmin-like protein 2	Q9HCH5
VCSMNVGNSLAKT	13	Zinc finger protein 548	Q8NEK5
NTKGGDAPAAGEDA	14	40S ribosomal protein S25	P62851
KVEQAVETEPEPEL	14	Apolipoprotein E	P02649
VLLAPIQYFIATKL	14	ATP-binding cassette sub-family C member 9	O60706
KDSPQTIPTYTDAL	14	Caspase-14	P31944
YMLNNISGDVLVAA	14	Dynein heavy chain 1, axonemal	Q9P2D7
GVVDSEDLPLNISR	14	Heat shock protein HSP 90-beta	P08238
GAAVAPEGNQKKKR	14	Isoform 4 of ATP synthase subunit s-like protein	Q9NW81-4
VNLSKANVDISAPK	14	Neuroblast differentiation-associated protein AHNAK	Q09666
FDEFFSEGCAPGSK	14	Serotransferrin	P02787
IKEGAAVIDVGINR	14	Bifunctional methylenetetrahydrofolate dehydrogenase/cyclohydrolase, mitochondrial	P13995
KRCAAACGACARPP	14	Flt3-interacting zinc finger protein 1	Q96SL8
HFDQCLMILNSPGN	14	cGMP-specific 3',5'-cyclic phosphodiesterase	O76074
MVGREYEAEGIAKD	14	Methylcrotonoyl-CoA carboxylase beta chain, mitochondrial	Q9HCC0
		Methylcrotonoyl-CoA carboxylase beta chain, mitochondrial	D6RD67
PPVCSKTIALPASA	14	Putative uncharacterized protein MGC39545	Q8IYB0
HVASVPAKLWARIV	14	Lethal(2) giant larvae protein homolog 1	Q15334
GEDIDTISPTLGFNI	15	ADP-ribosylation factor-like protein 2	P36404
PLPPGPAQASVALPP	15	Protein enabled homolog	Q8N8S7
TGDDKGESNDGKSKV	15	Tyrosine-protein phosphatase non-receptor type 11	Q06124
DPATTKDYVSADPGT	15	Protein PBMUCL2	E2RYF7
ALPLGPGASGLLTGT	15	Transmembrane protein 253	P0C7T8
QATEATIVTLDSDNI	15	Copper-transporting ATPase 1	Q04656
TLLIAVIVFGNVLVC	15	D(2) dopamine receptor	P14416
VSLATVIFVASQKAL	15	Integrator complex subunit 7	Q9NVH2
GRLLFGRIADYVPGV	15	Monocarboxylate transporter 10	Q8TF71
SYELPDGQVITIGNER	16	Actin, cytoplasmic 1	P60709

Sequence	Length	Source	Accession Number
TGAIVDVPVGEELLGR	16	ATP synthase subunit alpha, mitochondrial	P25705
ANGDGGMTTVQCPDGV	16	E3 ubiquitin-protein ligase MYCBP2	O75592
GGVAAGVAVLDNPYPV	16	Glutathione synthetase	P48637
LLGATCGLKTILTLTG	16	Glycerol-3-phosphate phosphatase	A6NDG6
SSQPLASKQEKDGTEK	16	Isoform HMG-R of High mobility group protein HMG-I/HMG-Y	P17096-3
RQEKKRATRQLLSALT	16	PH and SEC7 domain-containing protein 2	Q9H0X9
PTTPKKPAPKELAPTT	16	Proteoglycan 4	Q92954
AYSFKYLKNKPVKELR	16	Transcription elongation factor SPT6	Q7KZ85
TITLEVEPSDTIENVK	16	Ubiquitin-40S ribosomal protein S27a	P62979
PALPALAPLSSPAKTL	16	Vertnin	Q9H8Y1
KVIYPAVEGRIKFSTG	16	5'-nucleotidase	P21589
LQKVLEPTSTHESEHQ	16	Adhesion G protein-coupled receptor L1	O94910
AVAEPQIAMFCGKLNM	16	Amyloid-like protein 2	Q06481
GLVQALGAHLYQNVFAC	17	C-1-tetrahydrofolate synthase, cytoplasmic	F5H2F4
EVGQGNGLQKAQAHDGA	17	Pleckstrin homology domain-containing family M member 1	Q9Y4G2
RAVLLAGPPGTGKTALA	17	RuvB-like 1	Q9Y265
LVSLGALGSVEFSLATL	17	BPI fold-containing family B member 3	P59826
		Adhesion G-protein coupled receptor G4	Q8IZF6
VAPEEHPVLLTEAPLNPK	18	Actin, cytoplasmic 1	P60709
LSGFQARNALLQSNLSQT	18	Intersectin-2	Q9NZM3
PPARLVEVPAAPVRVVET	18	Ribosomal oxygenase 1	Q9H6W3
TPTATQVDGADLASPMSP	18	Ral GTPase-activating protein subunit alpha-1	H0YJB5
		Microtubule-associated serine/threonine-protein kinase 2	Q6P0Q8
VAPDEHPILLTEAPLNPK	18	Beta-actin-like protein 2	Q562R1
VKVHMNDNSTKSLMVDERQ	19	Amyloid beta A4 precursor protein-binding family B member 1-interacting protein	Q7Z5R6
LLTIVALAGATGLRDKAQE	19	Dynein assembly factor 5, axonemal	Q86Y56
LIPGKNPKIQNSNMPRECI	19	Guanylate-binding protein 4	Q96PP9
TKPKPTKRKRKGSSAVGSD	19	Integrator complex subunit 3	Q68E01
CKNKLFCVAELQLATTVSQ	19	Integrin alpha-E	P38570
VSGGKDGNGSSTSVQGSPAY	20	Baculoviral IAP repeat-containing protein 6	Q9NR09
TPQPAASLPDNTMVTHLFQK	20	Serine/threonine-protein phosphatase 6 regulatory subunit 2	O75170
QVGPEGILQNGAVDDSVAKTSQ	22	Regulator of nonsense transcripts 1	Q92900
LMLGGGGYTIRNVARCWTYETAVA	24	Histone deacetylase 2	Q92769
IDVSGPKVDVDIPDVNIEGPDAKL	24	Neuroblast differentiation-associated protein AHNAK	Q09666

[a] Peptides are presented in N-terminal to C-terminal orientation.

Table S3. HLA-F*01:04 restricted peptides[a].

Sequence	Length	Source	Accession Number
VISSIEQK	8	14-3-3 protein gamma	P61981
KVGDDIAK	8	60S ribosomal protein L12	P30050
SQKPVMVK	8	60S ribosomal protein L28	P46779
VVGAQSLK	8	6-phosphogluconate dehydrogenase, decarboxylating	P52209
KGLGAQKV	8	ADP-ribosylation factor GTPase-activating protein 2	Q8N6H7
MAHMASKE	8	Glyceraldehyde-3-phosphate dehydrogenase	P04406
AAQEEYVK	8	Isoform 2 of Fructose-bisphosphate aldolase A	P04075-2
NQELRQVK	8	Isoform 3 of Importin-5	O00410-3
SKEYFSKQ	8	Peroxiredoxin-1	Q06830
KSVQKIGA	8	Sodium-coupled neutral amino acid transporter 2	Q96QD8
SHEAEVLK	8	Stathmin	P16949
AQVRIGGK	8	Transcription factor BTF3	P20290
QALASGKI	8	Glyoxylate reductase/hydroxypyruvate reductase	Q9UBQ7
RGAARLVG	8	Helicase SKI2W	Q15477
SHQLVSLQ	8	Transcription factor SPT20 homolog-like 1	Q3ZLR7

Sequence	Length	Source	Accession Number
AGVKINPK	8	Glutamate dehydrogenas	B3KT18
AGKVNLPK	8	Retinal homeobox protein Rx	Q9Y2V3
		Zinc finger protein PLAGL1	Q9UM63
KQEIVAEK	8	Coronin-1C	Q9ULV4
KLSTSAPK	8	Calcium-independent phospholipase A2-gamma	Q9NP80
KGESLGIK	8	Cysteine and glycine-rich protein 1	P21291
KVAEAGVK	8	Transcription factor Sp5	Q6BEB4
NTGQRAVLK	9	40S ribosomal protein SA	P08865
AALLKASPK	9	60S ribosomal protein L14	P50914
KAEAQIAAK	9	Aspartate aminotransferase, mitochondrial	P00505
VNASASSLK	9	Fascin	Q16658
SLIGKKGQQ	9	Histone H2A.Z	P0C0S5
AVTKYTSAK	9	Histone H2B type 1-K	O60814
GSEIVVAGK	9	Inter-alpha-trypsin inhibitor heavy chain H2	P19823
AVELAANTK	9	Isoform 2 of Transketolase	P29401-2
STAAQQELR	9	Myosin-9	P35579
AIGSGTGIL	9	Protein transport protein Sec61 subunit alpha isoform 1	B4DR61
APNHAVVTR	9	Serotransferrin	P02787
NADELVKQK	9	T-complex protein 1 subunit alpha	P17987
TATQLAVNK	9	T-complex protein 1 subunit eta	Q99832
VDENGKISR	9	Ubiquitin-40S ribosomal protein S27a	P62979
IAGAVTGSL	9	Complex I assembly factor TIMMDC1, mitochondrial	Q9NPL8
RATVVESSEK	10	14-3-3 protein gamma	P61981
IAGQVAAANK	10	40S ribosomal protein S19	P39019
RTQAPTKASE	10	60S ribosomal protein L29	P47914
NLQTVNVDEN	10	60S ribosomal protein L31	P62899
AGFAGDDAPR	10	Actin, cytoplasmic 1	P60709
AATSEGVQVK	10	Methyl-CpG-binding protein 2	A0A087WXF0
DINTDGAVNF	10	Protein S100-A8	P05109
LSGVSSNIQK	10	Rho GTPase-activating protein 30	Q7Z6I6
DSGFQMNQLR	10	Serotransferrin	P02787
VADISGDTQK	10	Staphylococcal nuclease domain-containing protein 1	Q7KZF4
DVNAAIATIK	10	Tubulin alpha chain	F5H5D3
ISEQFTAMFR	10	Tubulin beta chain	P07437
LAVNMVPFPR	10	Tubulin beta chain	P07437
YQELQITAGR	10	Keratin, type II cytoskeletal 1b	Q7Z794
ANVGAGKKPKE	11	40S ribosomal protein S24	P62847
VLKAQAQSQKA	11	60S ribosomal protein L34	P49207
VTGGAASKLSK	11	60S ribosomal protein L35	P42766
AGEKVEKPDTK	11	60S ribosomal protein L6	Q02878
EITALAPSTMK	11	Actin, cytoplasmic 1	P60709
HQGVMVGMGQK	11	Actin, cytoplasmic 1	P60709
VSLLADCNDHQ	11	Armadillo repeat-containing protein 6	Q6NXE6
PEPAKSAPAPK	11	Histone H2B type 1-K	O60814
SSQPLASKQEK	11	Isoform HMG-R of High mobility group protein HMG-I/HMG-Y	P17096-3
NNLEALEDFEK	11	Lipocalin-1	P31025
GLSTESILIPR	11	Lipocalin-1	P31025
AAEIDEEPVSK	11	Nascent polypeptide-associated complex subunit alpha, muscle-specific form	E9PAV3
IVTDRETGSSK	11	Nucleolin	P19338
ATAVMPDGQFK	11	Peroxiredoxin-1	Q06830
SRMYDVLEPQQ	11	A-kinase anchor protein SPHKAP	Q2M3C7
TGETSRALSSSK	12	Activated RNA polymerase II transcriptional coactivator p15	P53999
YFPTQALNFAFK	12	ADP/ATP translocase 4	Q9H0C2
MKSMPPSLETSP	12	E3 ubiquitin-protein ligase MYCBP2	O75592
DAGTIAGLNVLR	12	Heat shock cognate 71 kDa protein	P11142
HAVSEGTKAVTK	12	Histone H2B	P58876
FVEGLPINDFSR	12	Isoform 3 of Malate dehydrogenase, cytoplasmic	P40925-3

Sequence	Length	Source	Accession Number
STDYGIFQINSR	12	Lysozyme C	P61626
MYLGYEYVTAIR	12	Serotransferrin	P02787
ESKDPADETEAD	12	Stathmin	P16949
GQGVHHTAGQVG	12	Suprabasin	Q6UWP8
QDALERALPELQ	12	Sterile alpha and TIR motif-containing protein 1	Q6SZW1
PQGCEQALRVTR	12	Potassium voltage-gated channel subfamily KQT member 2	A0A0D9SFE0
RMQPCGVDGDIV	12	Ninein-like protein	Q9Y2I6
NFDYSSLALLLQ	12	Liprin-alpha-2	O75334
NSTKLPSSDNLQ	12	Muscarinic acetylcholine receptor M3	P20309
SDTHFNGIVIMA	12	Olfactory receptor 5AP2	Q8NGF4
EVNSIGNHPQVQ	12	StAR-related lipid transfer protein 9	Q9P2P6
KQRLKDAGYGEK	12	Transcription initiation factor TFIID subunit 1-like	Q8IZX4
GNPTVEVDLFTSK	13	Alpha-enolase	P06733
TVLIMELINNVAK	13	ATP synthase subunit beta, mitochondrial	P06576
VTGYNDPETGNII	13	Desmoplakin	P15924
VNVDEVGGEALGR	13	Hemoglobin subunit beta	P68871
TLDDMRRLIDLGV	13	Isoform 2 of Lysine-specific demethylase 5B	Q9UGL1-2
QEKMTMNKELSPD	13	Isoform 2 of NAD kinase	O95544-2
ENAASGDAAVHQR	13	Regulation of nuclear pre-mRNA domain-containing protein 1A	Q96P16
KMGIEQNMCEVNR	13	Serine-protein kinase ATM	Q13315
MRAIRSVNPNLQN	13	E3 ubiquitin-protein ligase NRDP1	Q9H4P4
MMENYSSLVSLGL	13	Zinc finger protein 850	A8MQ14
NSIRDSLNEYEAK	13	Laminin subunit alpha-3	Q16787
TVWPDHGVPETTQ	13	Receptor-type tyrosine-protein phosphatase beta	P23467
VSCYIQNLLLGQE	13	Butyrophilin subfamily 1 member A1	Q13410
DATMNGTSSQPKK	13	Centrosomal protein of 128 kDa	Q6ZU80
AFELTVSCQGGLP	13	Melanocyte protein PMEL	P40967
KCKGQLLIFGATN	13	Protein RCC2	Q9P258
KCGKWVQLQLAES	13	Coiled-coil domain-containing protein 141	Q6ZP82
HQKDFASIVLLDQ	13	Putative ATP-dependent RNA helicase DDX12	Q92771
GSFPINGLHSHSE	13	Synaptotagmin-like protein 2	Q9HCH5
NTKGGDAPAAGEDA	14	40S ribosomal protein S25	P62851
FTQAGSEVSALLGR	14	ATP synthase subunit beta, mitochondrial	P06576
STGGAPTFNVTVTK	14	Profilin-1	P07737
FDEFFSEGCAPGSK	14	Serotransferrin	P02787
SVIPSDGPSVACVK	14	Serotransferrin	P02787
EDPQTFYYAVAVVK	14	Serotransferrin	P02787
QDGSCNGLQHYAAL	14	DNA-directed RNA polymerase, mitochondrial	O00411
QNADSCEICCLVLR	14	Zinc finger protein 211	Q13398
PHAQLARSPKESPA	14	Inositol hexakisphosphate kinase 3	Q96PC2
MVGREYEAEGIAKD	14	Methylcrotonoyl-CoA carboxylase beta chain, mitochondrial	Q9HCC0
TWCAACTVPSPQQV	14	UPF0606 protein KIAA1549	Q9HCM3
VSASASPCLHPGAQ	14	Tubulin polyglutamylase TTLL5	Q6EMB2
SVTSAQMLQGCTIF	14	Insulin-like growth factor 1 receptor	P08069
RSLLTGENHFQTVQ	14	Probable E3 ubiquitin-protein ligase DTX3	H0YHF9
		40S ribosomal protein S21	Q13666
		DnaJ homolog subfamily B member 9	Q9UBS3
EKSATCSNEEKDNL	14	Protein kintoun	Q9NVR5
FSWLSAAPTSLGPR	14	Myeloid cell surface antigen CD33	P20138
ENTLYSNDNGSNLQ	14	Serum response factor-binding protein 1	Q8NEF9
AVMEKNPHTADAQQ	14	Probable ribonuclease ZC3H12B	Q5HYM0
KMCSPGQQSFMLLV	14	Ankyrin repeat and SOCS box protein 9	R4GN94
IRALINNSGNATFY	14	Glutamate-rich protein 6B	Q5W0A0
HFDQCLMILNSPGN	14	cGMP-specific 3',5'-cyclic phosphodiesterase	O76074
GALQNIIPASTGAAK	15	Glyceraldehyde-3-phosphate dehydrogenase	P04406
AMGIMNSFVNDIFER	15	Histone H2B	P58876
AVFVDLEPTVIDEVR	15	Tubulin alpha chain	F5H5D3
AILVDLEPGTMDSVR	15	Tubulin beta chain	P07437

Sequence	Length	Source	Accession Number
SQETPATKKAVQGGG	15	U1 small nuclear ribonucleoprotein A	P09012
QEMTFGAQASVATEP	15	Coiled-coil domain-containing protein 17	Q96LX7
NPSAALSGSQILNKN	15	Double-stranded RNA-binding protein Staufen homolog 1	O95793
RTSSMANISMCQALG	15	Major facilitator superfamily domain-containing protein 8	Q8NHS3
EECGKAFSQSSILTT	15	Zinc finger protein 431	Q8TF32
DINSSMTNSTAASRP	15	Poly(rC)-binding protein 1	Q15365
KLQVLDLRNVDENFC	15	PRAME family member 8	Q5VWM4
		PRAME family member 7	Q5VXH5
ISRTVGRVAGGIQFL	15	LHFPL tetraspan subfamily member 6 protein	Q9Y693
GFMASFLDFLKSGKR	15	Proline-rich protein 12	Q9ULL5
GDNSGNGTQEKIAEG	15	Type II iodothyronine deiodinase	Q92813
GQPMSCTCGPLRSLT	15	Zinc transporter 3	H7BZ83
SYELPDGQVITIGNER	16	Actin, cytoplasmic 1	P60709
TGAIVDVPVGEELLGR	16	ATP synthase subunit alpha, mitochondrial	P25705
ETERLPGAQGPCSAVS	16	Tubulin beta-4A chain	M0QZL7
TITLEVEPSDTIENVK	16	Ubiquitin-40S ribosomal protein S27a	P62979
QGRAAPAFKGGGGPRS	16	BAH and coiled-coil domain-containing protein 1	Q9P281
QRDQSEPLGRVLSRIA	16	2',5'-phosphodiesterase 12	Q6L8Q7
VLPAPLSAPLSAPQAV	16	MAGE-like protein 2	Q9UJ55
CRLPKRQCNRHYCWEK	16	CXXC-type zinc finger protein 1	Q9P0U4
FSSPATSRNLVLTPVN	16	GPI mannosyltransferase 4	Q86VD9
FHVLKMLLNLSENPAV	16	G-protein coupled receptor-associated sorting protein 2	Q96D09
APDSAKTKALQTVIEM	16	ELKS/Rab6-interacting/CAST family member 1	Q8IUD2
KTNKSMYECKKSDQYD	16	MAGUK p55 subfamily member 7	Q5T2T1
KLSTLTSCVHASCALL	16	DENN domain-containing protein 1C	Q8IV53
NALARYASICQQNGLVP	17	Fructose-bisphosphate aldolase B	P05062
SGALNEALLSSKDDVGK	17	Isoform 4 of Myosin-14	Q7Z406-4
LVANELDAGRVQLLLAQ	17	Putative uncharacterized protein FLJ31958	Q96MT0
AWRAASAVGATAVETSR	17	Ankyrin repeat domain-containing protein SOWAHD	A6NJG2
ATKAAPGDAGLQGGAGC	17	Isoform 4 of Trafficking protein particle complex subunit 6A	O75865-4
GKGVGLRGQLTKKPLQL	17	Serine incorporator 4	A6NM42
		FK506-binding protein 1	Q5T1M5
VAPEEHPVLLTEAPLNPK	18	Actin, cytoplasmic 1	P60709
VAPDEHPILLTEAPLNPK	18	Beta-actin-like protein 2	Q562R1
SKTRREMQSVVQLIMTRD	18	ESF1 homolog	Q9H501
FVAAIPLLALAGALALRN	18	Molybdate-anion transporter	Q6N075
LCNSSESTSHPELQAPPN	18	Protocadherin gamma-B5	Q9Y5G0
AIAELGIYPAVDPLDSTSR	19	ATP synthase subunit beta, mitochondrial	P06576
REANALAMQQKWNSLDEGR	19	Roquin-2	Q9HBD1
FPNKVDFIQELSEVLVQFG	19	DENN domain-containing protein 5B	Q6ZUT9
FFSITRAAPGARWGQQAHS	19	Ectonucleoside triphosphate diphosphohydrolase 6	O75354
KMAVLANGVMNSLQDRYGSY	20	ADP-ribosylation factor GTPase-activating protein 2	Q8N6H7
VEGDNIYVRHSNLMLEIDGG	20	Band 4.1-like protein 2	E9PPC9
PTATNQQNKRNQCSCNVTEF	20	Proto-oncogene tyrosine-protein kinase ROS	P08922
		Tyrosine-protein kinase receptor	Q5H8Y1
PLCISGIVQRVNETQNGTNN	20	ATP-binding cassette sub-family C member 9	O60706
TCFATKNGIKVTVENAKCVQ	20	Cell cycle checkpoint protein RAD1	O60671
DLYANTVLSGGTTMYPGIADR	21	Actin, cytoplasmic 1	P60709
FTESTTSDASEHASQSVPMVT	21	Nucleoprotein TPR	P12270

[a] Peptides are presented in N-terminal to C-terminal orientation.
